# Benchmark competitiveness and generalization are not equivalent: a critical commentary on HybridSP

**DOI:** 10.1093/bib/bbag193

**Published:** 2026-04-25

**Authors:** Marcio Madrid, Melania Madrid, Isaac Zablah

**Affiliations:** Faculty of Medical Sciences, National Autonomous University of Honduras, Calle la Salud, 11101, Tegucigalpa, Honduras; Faculty of Biology, University of Salamanca, Calle Donantes de Sangre, 37007, Salamanca, Spain; Neurosciences Research Group, Faculty of Medical Sciences, National Autonomous University of Honduras, Calle la Salud, 11101, Tegucigalpa, Honduras

**Keywords:** protein–ligand scoring, statistical potentials, deep learning, virtual screening, benchmark bias, out-of-distribution generalization, data leakage

## Abstract

This Letter comments on the article by Wang *et al.* on HybridSP and argues that strong benchmark performance should not be interpreted as direct evidence of robust generalization. While the model is interpretable, computationally efficient, and methodologically valuable, its reported results may be inflated by benchmark-specific coefficient tuning and by comparisons across scoring pipelines with different underlying tasks. We further note that performance drops on bias-reduced and more challenging datasets already suggest limits to transferability. We therefore advocate for stricter validation frameworks to support broader claims regarding generalization in protein–ligand scoring.

To the Editor,

We read with considerable interest the case study by Wang *et al.* [1], which revisits the long-standing debate between physically grounded statistical potentials and data-driven deep learning (DL) approaches in protein–ligand scoring. The authors introduce HybridSP a hybrid model combining distance-dependent atom–atom, atom–residue, and orientation-dependent potentials and report a 91.6% docking success rate alongside an enrichment factor (EF) of 29.35 at the top 1% on CASF-2016, performances they describe as rivaling or surpassing state-of-the-art DL models. The contribution is timely and welcome: in a field that too readily equates architectural complexity with scientific progress, a well-designed interpretable model that recovers competitive performance represents a genuine methodological advance.

Our primary concern, however, relates to the relationship between benchmark performance and genuine generalization. A subtle but consequential issue deserves explicit attention. The weighting coefficients of HybridSP (a, b, c) for each application variant were docking-oriented, screening-oriented, and balanced. Them were tuned specifically for performance on CASF-2016 (Eq. 8 and surrounding discussion in [1]). Because CASF-2016 simultaneously serves as the tuning criterion and the primary evaluation benchmark, the reported metrics likely overestimate the model’s true generalization capacity. This is a form of criterion leakage that is distinct from simple train–test overlap, and it merits transparent discussion. A rigorous evaluation would require these coefficients to be fixed on a held-out validation set prior to any CASF-2016 assessment.

A related methodological concern involves the comparability of the scoring pipelines evaluated. HybridSP operates within a traditional “dock-then-score” framework, where binding poses are first generated by AutoDock Vina and subsequently re-ranked. Several of the DL baselines selected for comparison, such as RTMScore and GenScore, are also re-scoring functions applied to Vina-generated poses; however, others (including AlphaFold3 variants) perform end-to-end co-folding and pose prediction. Comparing these two fundamentally different paradigms on a single success-rate metric conflates the quality of pose generation with the quality of scoring, introducing an asymmetry that can systematically favor or penalize either approach depending on the target and benchmarking protocol. A stratified analysis distinguishing between scoring functions and full-pipeline models would substantially strengthen the interpretive clarity of these comparisons.

It is also worth noting that the paper’s own results already counsel measured expectations. Performance decreases on the bias-reduced DUD-AD benchmark compared with DUD-E, a pattern consistent with prior findings that highlight the degree to which DUD-E-based EFs can reflect dataset-specific regularities rather than transferable recognition of binding interactions [2]. Similarly, both HybridSP and the DL comparator RTMScore show reduced accuracy on the flexible P450 enzyme set, a result the authors attribute to pose inaccuracies and dataset bias [1]. On the free energy perturbation (FEP) dataset, HybridSP trails established FEP-based approaches in terms of affinity reproduction [1]. These findings, taken together, suggest that the model is benchmark-competitive and mechanistically informative, but they do not yet constitute evidence of superiority over DL in a broader or transferable sense.

A stronger claim of generalizability would require validation schemes designed explicitly to probe performance beyond the established benchmark ecosystem. Specifically, we would encourage the authors to consider: (i) target-family holdout evaluations, in which protein families represented in the training data are entirely excluded from testing; (ii) scaffold-clustered or chronological splits to reduce chemotype overlap between training and test sets; (iii) cross-docking protocols to assess robustness to receptor conformational variability; and (iv) prospective or semi-prospective screening against experimentally confirmed inactive compounds not present in curated decoy libraries. Such experiments are demanding, but they constitute the evidentiary standard necessary to support the translational applicability of these tools in practical drug discovery pipelines, including in resource-limited research environments where access to large-scale DL infrastructure is constrained [3–5].

We also invite the authors to clarify the epistemological status of the affinity-weighted training scheme. The incorporation of experimental binding affinities as weighting factors during statistical model construction introduces a supervised signal that partly bridges the conceptual boundary between knowledge-based and learning-based approaches. While this is a methodologically interesting design choice and clearly beneficial in practice, as the results suggest, it complicates straightforward comparison with truly unsupervised statistical potentials and warrants transparent framing in the discussion.

None of these observations diminish the value of Wang *et al.*’s contribution. HybridSP is interpretable, computationally efficient, and physically grounded properties that are increasingly recognized as critical for scientific trust and for deployment in settings where black-box predictions are insufficient. The author’s central thesis, that well-designed statistical potential remains serious competitors to DL-based scoring functions, stands on solid ground. We argue, however, that this claim is more compelling and more durable when supported by validation schemes that go beyond established benchmark ecosystems. We hope these observations contribute constructively to an ongoing and important methodological conversation.

Key Points

- Competitive performance on established benchmarks does not, by itself, demonstrate out-of-distribution generalization.
- Tuning HybridSP coefficients on CASF-2016 and evaluating on the same benchmark may overestimate its true generalizability.
- Comparisons between re-scoring models and end-to-end structure prediction pipelines should be interpreted cautiously because they address different tasks.
- Results on bias-reduced and more challenging datasets already indicate that benchmark success is not fully equivalent to transferable performance.
- More rigorous validation strategies, including target-family holdouts, scaffold-based splits, and prospective screening, are needed before broad claims of superiority can be sustained.

## Data Availability

No new data was generated or analyzed for this correspondence.

## References

[1] Wang Z, Wang S, Guo J et al. Could statistical potential models achieve comparable or better performance than deep learning models? *Brief Bioinform* 2026;27:bbag088. 10.1093/bib/bbag088 41766645 PMC12951076

[2] Chen L, Cruz A, Ramsey S et al. Hidden bias in the DUD-E dataset leads to misleading performance of deep learning in structure-based virtual screening. *PloS One* 2019;14:e0220113. 10.1371/journal.pone.0220113 31430292 PMC6701836

[3] Buttenschoen M, Morris GM, Deane CM. PoseBusters: AI-based docking methods fail to generate physically valid poses or generalise to novel sequences. *Chem Sci* 2024;15:3130–9. 10.1039/D3SC04185A 38425520 PMC10901501

[4] Masters MR, Mahmoud AH, Lill MA. Investigating whether deep learning models for co-folding learn the physics of protein-ligand interactions. *Nat Commun* 2025;16:8854. 10.1038/s41467-025-63947-5 41053181 PMC12501370

[5] Gu S, Shen C, Zhang X et al. Benchmarking AI-powered docking methods from the perspective of virtual screening. *Nat Mach Intell* 2025;7:509–20. 10.1038/s42256-025-00993-0

